# Tropical rain forest evolution: palms as a model group

**DOI:** 10.1186/1741-7007-11-48

**Published:** 2013-04-15

**Authors:** Thomas LP Couvreur, William J Baker

**Affiliations:** 1 Institut de Recherche pour le Développement (IRD), UMR DIADE, 911, avenue Agropolis, BP 64501, F-34394 Montpellier cedex 5, France; 2 Université de Yaoundé I, Ecole Normale Supérieure, Département des Sciences Biologiques, Laboratoire de Botanique systématique et d'Ecologie, B.P. 047, Yaoundé Cameroon; 3 Royal Botanic Gardens, Kew, Richmond, Surrey, TW9 3AB, United Kingdom

## 

The biological diversity and complexity of tropical rain forests have fascinated biologists for centuries. This, the most species-rich terrestrial biome on Earth, is now severely threatened by human activity and yet we are still far from answering fundamental questions about its origins and evolution. Where and how did this huge diversity originate? What evolutionary processes were involved? How can we explain the patterns of current biodiversity across this biome?

Basic biodiversity data, such as species composition, distribution and phylogenetic relationships, are fundamental prerequisites for a better understanding of tropical rain forest evolution. Species inventories, for example, can be exploited to unpick the evolutionary history of tropical rain forest assemblages using community phylogenetic approaches [1]. Perpendicular to this approach, taxonomic and systematic research focusing on lineages predominantly restricted to tropical rain forests can be rewarding [2]. In particular, dated phylogenies of tropical rain forest lineages can be used to unravel the evolutionary origins, timescales and processes of this biome as a whole. In this comment, we describe how one such tropical rain forest-restricted lineage, the palm family, has provided evidence of global rain forest history in a succession of recent studies integrating a range of basic biodiversity datasets [3-9], the first of which was published in *BMC Biology *[3], and now represents a model for the study of the tropical rain forest biome that may, potentially, be applied to other lineages of organisms.

## Identifying model groups for tropical rain forest research

**Figure 1:**
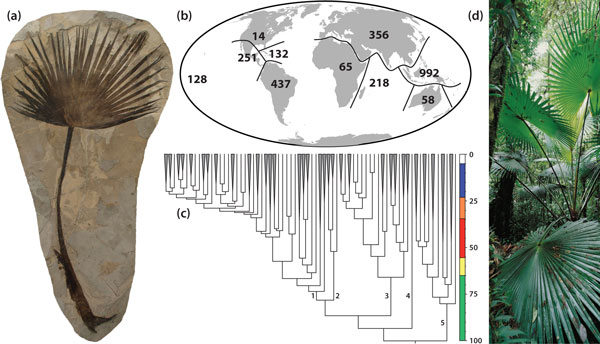
**Palms as a model group for tropical rain forest research**. **(a) **Palms have a well-documented fossil record dating back to the Late Cretaceous - for example, *Sabalites *leaf fossil, Eocene, Wyoming (image: Board of Trustees, National Museums Liverpool, Liverpool, UK). **(b) **Palm species taxonomy and distribution is well understood - species richness derived from [15] with updates. **(c) **A global, dated phylogeny is available [7,13] (branch numbers indicate palm subfamilies: (1) Arecoideae, (2) Ceroxyloideae, (3) Coryphoideae, (4) Nypoideae, (5) Calamoideae). Blue = Miocene, Orange = Oligocene, Red = Eocene, Yellow = Palaeocene, Green = Cretaceous. **(d) **More than 90% of all palms occur in tropical rain forest - for example, *Saribus papuanus *in New Guinea (image: A McRobb, RBG Kew).

Simple criteria can be used to identify lineages most suitable for the study of biome evolution and ecology: 1) the lineage should be ecologically representative of the biome under investigation, 2) species taxonomy and distributions of the lineage should be well-documented, 3) comprehensive phylogenetic hypotheses of the lineage should be available, and 4) the historical timeframe of the lineage should coincide with that of the biome (as indicated by the fossil record, for example). The palms (Arecaceae or Palmae) meet all of these criteria for tropical rain forests and are thus a good model group for that biome (Figure 1). Firstly, they are restricted almost entirely to the tropics and subtropics, with >90% of palm species diversity concentrated in tropical rain forest [3] where they play a prominent role in the vegetation, often as keystone species [10]. Indeed, fundamental structural properties render palms intolerant to cold [11], the primary factor determining their latitudinal limits [5]. Secondly, the taxonomy of palm species is well-known relative to other flowering plant families of comparable size (approximately 2,500 species in 184 genera) and is conveniently summarized in a global checklist with distributional data [12]. Thirdly, a complete phylogeny of all genera is available [13], now in time-calibrated chronogram form [3]. Finally, palms also have a long and well-documented fossil record [14] dating back to the Late Cretaceous (Turonian; 89.3 to 93.5 million years ago (Ma)) that spans the recorded history of the tropical rain forest biome [7]. In addition, a recent comprehensive family monograph provides biological information for all genera [15], and copious occurrence data are available from herbarium records and field surveys (for example, [10]). In summary, no larger plant tropical family is better served with basic biodiversity data than palms. The application of these criteria may help to identify other suitable model plant and animal lineages for tropical rain forest research [16-19].

## Origins of tropical rain forests

These resources have yielded details of palm evolutionary dynamics that shed light on the origins of tropical rain forests. We mined the palm fossil record for calibration points for molecular dating of the global genus-level palm phylogeny [13]. The resulting dated phylogeny and reconstructions of biogeographic history [3,7] indicate that extant palm lineages initially diversified during the mid-Cretaceous around 100 Ma at northern latitudes in the supercontinent of Laurasia. Importantly, we also demonstrated that palms underwent this early diversification in a tropical rain forest environment. The implication of these results is that tropical rain forest, or at least a tropical rain forest-like biome, existed at northern latitudes in Laurasia in the mid-Cretaceous, a controversial finding because the most widely accepted fossil evidence for the earliest tropical rain forest is post-Cretaceous, dating from the Palaeocene (55 to 65 Ma) within its current equatorial range [20,21]. Though our inferences may be met with scepticism, they are consistent with limited evidence of tropical rain forest-like fossil floras in the mid-Cretaceous of North America [22], as well as indirect evidence from molecular dating of other important major tropical lineages [16,17,23]. While mid-Cretaceous Laurasia may not have supported modern tropical rain forest as we know it today in terms of composition and diversity, the evolutionary histories captured in multiple tropical rain forest-restricted lineages suggest that the tropical rain forest biome, or something very close to it, originated well before the end of the Cretaceous (65 Ma). This implies that tropical rain forests are not only the most species-rich but also one of the oldest biomes on Earth.

The major dispersal routes of palms since the mid-Cretaceous inferred from the dated phylogeny [7] have further implications for tropical rain forest history. Palms migrated from Laurasia into their present day distribution in South America, Africa, and South-East Asia before the end of the Cretaceous, events that are consistent with the first occurrences of tropical rain forest fossil floras in those regions [20,21,24]. This adds weight to what may be a general pattern of northern latitude origins for tropical rain forest lineages with subsequent (Late Cretaceous or Cenozoic) migrations towards equatorial latitudes [21]. The absence of fossil evidence of equatorial tropical rain forest before this time lends support to this trend.

Both direct (fossil record) and indirect (molecular dating, biogeographic reconstruction) approaches to tropical rain forest history have limitations. Fossil floras provide the only direct evidence of past tropical rain forest extent, but are contingent on the distribution of sampling sites, fossil identification and the vagaries of stratigraphy. Molecular dating and biogeographic analyses offer a silver bullet, but rely on numerous methodological assumptions and uncertainties in calibration, and can yield wide confidence intervals. In our view, the two should be taken together to achieve a balanced view of biome history. The future is bright - phylogenetic methods and data are improving, and the fossil record remains a rich information source, especially for the palms. Rather than rejecting indirect findings as inconsistent with 'hard' fossil evidence, perhaps controversial results, such as ours, can be used to direct new research on the tropical rain forest fossil record.

## Modes of tropical rain forest diversification

As well as illuminating individual branching events, comprehensive dated phylogenies can provide a picture of overall lineage diversification processes across the timescale of an entire tree. We found that palm lineages have accumulated at a steady rate, at least until the end of the Oligocene (24 Ma), a pattern recovered in other tropical rain forest plants (for example, [16]) but also in tropical rain forest-restricted insect lineages (for example, [18]). This pattern supports the so-called 'museum model' of tropical rain forest diversification [25], which posits that long-term biome stability has permitted a constant build-up of diversity over time. The extent to which this is a general pattern is unclear. Indeed, it is well known that tropical rain forests have fluctuated over time and cannot be regarded as stable over 100 million year timescales. Other studies have revealed different pictures. For example, recent rapid diversifications have been discovered in certain tropical rain forest tree lineages [26] and in the family Menispermaceae a burst of speciation was detected after the end of the Cretaceous [17]. Even in the case of the palms, significant increases in diversification rate are required to reach modern levels of species diversity. Indeed, shifts to higher rates of diversification concentrated in more recent time frames (Miocene, 23 to 5 Ma) have been identified in several species-rich palm lineages [8].

Focused species-level studies will be required to build a clearer picture of tropical rain forest diversification modes, but for palms such studies are largely lacking at this time. Some authors have already underlined that at a regional scale palm diversification might not be constant, diversity being shaped by geological and climatic events [27], which may not be reflected in global studies [3]. Nevertheless, the 'big picture' studies of palms merit attention because they provide evidence spanning the full history of the tropical rain forest biome, unlike studies at lower taxonomic levels, which tend to be limited to recent timeframes and often emphasize rapid radiations. Most importantly, the palm studies indicate that museum model processes account for at least a proportion of tropical rain forest diversity, albeit in combination with other modes of diversification.

## Patterns of tropical rain forest diversity

Although palms are widespread in the tropics and subtropics, the distribution of major lineages and species diversity is highly structured geographically. The relative importance of environmental and historical factors in determining these patterns has been explored using a blend of macroecological and phylogenetic approaches. Using macroecological methods [5], palm species richness has been shown to be strongly determined by contemporary climatic variables, but the influence of Quaternary climate as well as deeper time biogeographic processes has also been highlighted. By combining distribution data with information from the dated phylogeny [4], a strong imprint on phylogenetic relatedness of geographic isolation and *in situ *diversification has been found, especially in South America and palm-rich islands such as Madagascar, which is indicative of strong dispersal limitation, a pattern also recovered in a further study focused on the Neotropical palms [9].

The most striking feature of diversity patterns in palms is the depauperate African palm flora, which comprises just 65 species, almost seven times fewer than South America and more than 15-fold fewer than the Malesian archipelago. This phenomenon has been explained by extinction due to significant loss of tropical rain forest through time linked to climate change [28]. Here, studies produce divergent results. One study [4] finds support for this hypothesis whereas another [8] suggests that diversification rate increases in American and South East Asian lineages, rather than decreases in African lineages, may account for the disparity, perhaps due to major tectonic processes in these regions. In fact, the two explanations may not be in conflict and it is reasonable to accept that both processes may be involved. The factors affecting palm diversity patterns highlighted by these studies - modern and historical climate, climate change, dispersal limitation, tectonic history and deep biogeographic history - are likely to be relevant to diversity patterns across tropical rain forest biota.

## Conclusion

The availability of a critical mass of biodiversity data has resulted in palm research that provides new insights into the global evolution and ecology of tropical rain forest. Basic biodiversity research that addresses the open questions 'where do species occur and how are they related?' must remain a priority for universities, institutions and funding agencies (private and national) in the years to come. Without such fundamental work, we will never fully understand the factors that have shaped Earth's biodiversity and how these may determine its future. In turn, this also requires proper funding and maintenance of the world's biodiversity repositories, herbaria and museums, which provide so much data for this research.

Single-lineage studies provide valuable insights into the evolutionary history of tropical rain forests, but remain limited in their explanatory power when comparable studies of other groups are lacking. Although methodological and data challenges remain, the integration of phylogenetic, biogeographic, macroecological and diversification studies across numerous tropical rain forest-restricted lineages represents our best hope for understanding the global evolution and ecology of the tropical rain forest biome. These studies should be viewed as complementary to direct evidence that we gain from the fossil record. Such an approach has already been taken for the diversification history of the Neotropics [29], and might effectively be extended to target tropical rain forest globally or indeed any of Earth's megadiverse biomes.

## Note

This article is part of the *BMC Biology *tenth anniversary series. Other articles in this series can be found at http://www.biomedcentral.com/bmcbiol/series/tenthanniversary.
